# An update to the taxonomy of the genus *Gastroserica* Brenske (Coleoptera, Scarabaeidae, Sericini)

**DOI:** 10.3897/zookeys.426.7578

**Published:** 2014-07-17

**Authors:** Wan-Gang Liu, Ming Bai, Xing-Ke Yang, Dirk Ahrens

**Affiliations:** 1 Key Laboratory of Zoological Systematics and Evolution, Institute of Zoology, Chinese Academy of Sciences, Box 92, No. 1, Beichen West Road, Chaoyang District, Beijing, 100101, P.R. China; 2 University of Chinese Academy of Sciences, Yuquan Road, Shijingshan, Beijing, 100039, P.R. China; 3 Zoologisches Forschungsmuseum A. Koenig, Adenauerallee 160, 53113 Bonn, Germany

**Keywords:** Beetles, Melolonthinae, chafers, *Gastroserica*, China, Laos, new species

## Abstract

Based on the examination of newly collected material and additional specimens housed in Chinese collections, our knowledge of *Gastroserica* Brenske, 1897, is expanded. Here, seven new species are described, including habitus photographs and illustrations of the male genitalia: *Gastroserica haoyui* **sp. n.** (China: Zhejiang Prov.), *G. fengduana* **sp. n.** (China: Sichuan Prov.), *G. wenzhui* **sp. n.** (China: Guangxi Prov.), *G. damingshanica* **sp. n.** (from China: Guangxi Prov.), *G. jinxiuensis* **sp. n.** (China: Guangxi Prov.), *G. liboensis* **sp. n.** (China: Yunnan Prov.) and *G. carolusi* **sp. n.** (Laos). Additionally, we provide a distribution map of the new taxa and new distribution records of the known taxa.

## Introduction

The species of the genus *Gastroserica* Brenske, 1897, from the Asiatic mainland were last revised by Ahrens (2000). Since then, ten additional species have been described (Ahrens 2002, 2007; Ahrens and Pacholátko 2003; Liu et al. 2011), bringing the total to 37, all occurring in East and Southeast Asia.

The genus *Gastroserica* was established by Brenske (1897) based on characters of the antenna (club with four antennomeres in males, but with three or four in females) and the anterior angles of pronotum (obsolete). In addition to this, the genus is also defined by the flat mentum, the carinate and ventrally produced hypomeron, as well as a long and apically produced pygidium, which is not completely covered by the elytra. Due to its hypothesized sister relationship to the genus *Neoserica* Brenske, 1894, sensu stricto (Liu et al., unpublished DNA data), the generic diagnosis should include the absent continuous serrated line adjacent to the anterior margin of the metafemur and to the dorsal margin of metatibia.

In this paper, we review material deposited in Chinese institutional collections and some recently collected material received from various European collections. We describe six new taxa from China and one from Laos. A distribution map for the new taxa is presented and new distribution data are given for the known species.

## Material and methods

The terminology and methods used for measurements, specimen dissection and genital preparation follow Ahrens (2004). Data from specimens examined is cited in the text with original label contents given in quotation marks, multiple labels are separated by a “/”. Male genitalia were glued to a small pointed card attached to the specimen. Descriptions and illustrations of new taxa are based on the holotype specimen, while the variation of other specimens is given separately under variation. All descriptions and measurements were made under an Olympus SZX 12 microscope, and all genital and habitus illustrations were made with a digital camera (AxioCam HRc) attached to a stereo microscope (Zeiss Stereo Discovery V20) and Axio Version 4.8 software. Based on geographical coordinates obtained from the labels and Google map, the distribution map was generated using Q-GIS 2.0.1 and Adobe Photoshop CS4.

Currently, most female specimens of Sericini are difficult to identify (to genus and species), as diagnostic characters of genera, as defined currently, often exhibit strong sexual dimorphism (e.g. the number of antennomeres of club). In *Gastroserica*, this is true for all known species. Although female genitalia may offer a sufficient number of sclerotised structures (Ahrens 2000) to infer species identity, this character trait is difficult to use. Often very similar species, which cannot be distinguished by external morphology due to their considerable variation in shape and colour, co-occur synoptically in many regions. Furthermore, the preparation of female genitalia requires a considerable amount of time, and is therefore not very suitable to the examination of large series of specimens, and many species are known only from male specimens. We therefore decided to study female morphology at a later stage, hopefully when barcoded female specimens can be unambiguously matched with male specimens (as has already been implemented for larval taxonomy: Ahrens et al. 2007; Sipek and Ahrens 2011).

Shape of aedeagus, particularly the parameres, offers sufficiently detailed and stable characters to allow discrimination of the species, while characters useful in other scarab groups such as shape of head and pronotum or the punctuation, colour and sculpture of the body surface are often highly variable in Sericini. This is particularly true in the genus *Gastroserica*. For this reason, we made no attempt to update the identification keys of Ahrens (2000) or Liu et al. (2011), which would, by necessity, have to be based nearly exclusively on male genital characters. In fact, we experienced from communication with other colleagues that illustrations of genital characters are much more straightforward than keys (in particular in species rich groups) in correct identification of species, which implies of course, that reliable species identification is impossible without examination of male genitalia.

Type specimens and additional material examined are deposited in the following institutions:

CAU Department of Entomology, China Agricultural University, Beijing (China);

CP Coll. Petr Pacholátko, Brno (Czech Republic);

HBUM Museum of Hebei University, Baoding (China, Hebei Prov.);

IZAS Institute of Zoology, Chinese Academy of Sciences, Beijing (China);

LSSYU Guangzhou, College of Life Sciences, Sun Yat-sen University, Guangzhou (China, Guangdong Prov.);

NMPC National Museum Prague (Natural History) (Czech Republic);

ZFMK Zoologisches Forschungsinstitut und Museum A. Koenig, Bonn (Germany).

## Taxonomy

### *Gastroserica* Brenske, 1897

*Gastroserica* Brenske, 1897: 412 [type species *Serica marginalis* Brenske, 1894, by subsequent designation (Nomura 1973)].

### Checklist of the known species:

*Gastroserica angustula* Brenske, 1897

*Gastroserica asulcata* Ahrens, 2000

*Gastroserica bicolor* Niijima & Kinoshita, 1923

*Gastroserica bilyi* Ahrens, 2000

*Gastroserica brevicornis* (Lewis, 1895)

*Gastroserica contaminata* Ahrens & Pacholátko, 2003

*Gastroserica dembickyi* Ahrens, 2000

*Gastroserica fanjingensis* Ahrens, 2000

*Gastroserica gemellata* Ahrens & Pacholátko, 2007

*Gastroserica guangdongensis* Ahrens, 2000

*Gastroserica guizhouana* Ahrens, 2000

*Gastroserica haucki* Ahrens, 2000

*Gastroserica herzi* (Heyden, 1887)

syn *Microserica hertzi* Reitter, 1896

*Gastroserica higonia* (Lewis, 1895)

*Gastroserica huaphanensis* Ahrens & Pacholátko, 2003

*Gastroserica hubeiana* Ahrens, 2000

*Gastroserica impressicollis* (Fairmaire, 1891)

*Gastroserica kabakovi* Ahrens, 2002

*Gastroserica kucerai* Ahrens & Pacholátko, 2003

*Gastroserica marginalis* (Brenske, 1894)

syn *Gastroserica puncticollis* Brenske, 1897

*Gastroserica mausonensis* Ahrens, 2000

*Gastroserica namthana* Ahrens, 2000

*Gastroserica napolovi* Ahrens, 2000

*Gastroserica nigrofasciata* Liu, Ahrens, Bai & Yang, 2011

*Gastroserica nikodymi* Ahrens, 2000

*Gastroserica patkaiensis* Ahrens, 2000

*Gastroserica pickai* Ahrens, 2000

*Gastroserica roessneri* Ahrens, 2000

*Gastroserica shaanxiana* Ahrens & Pacholátko, 2003

*Gastroserica sichuana* Ahrens, 2000

*Gastroserica stictica* Ahrens & Pacholátko, 2003

*Gastroserica sulcata* Brenske, 1897

*Gastroserica trilineata* Ahrens, 2000

*Gastroserica vinhphuensis* Ahrens, 2000

*Gastroserica viridis* Ahrens, 2000

*Gastroserica yingi* Ahrens & Pacholátko, 2007

*Gastroserica yunnanensis* Liu, Ahrens, Bai & Yang, 2011

### New species descriptions

#### Gastroserica carolusi

Taxon classificationAnimaliaColeopteraScarabaeidae

Liu & Ahrens sp. n.

http://zoobank.org/2D8DD31A-4E0D-4D47-82E9-5C0239A6ACE9

Figs 1A–D , 4

##### Material examined.

Holotype: ♂ “ NE-Laos: Hua Phan prov., Ban Saleui, Phou Pan (Mt.) - 20°12'N, 104°01'E; 11.iv.–15.v.2012; 1300-1900m; leg. C. Holzschuh Ankauf ZFMK Bonn 2012” (ZFMK). Paratypes: 135 ♂♂ “NE-Laos: Hua Phan prov., Ban Saleui, Phou Pan (Mt.) - 20°12'N, 104°01'E; 14.iv.–15.v.2012; 1300–1900m; leg. C. Holzschuh Ankauf ZFMK Bonn 2012/13” (ZFMK), 1 ♂ “NE-Laos: Hua Phan prov., Ban Saleui, Phou Pan (Mt.) ~20°12'N, 104°01'E; 1300–1900m; 01.–31.V.2011; leg. C. Holzschuh Ankauf ZFMK Bonn 2011” (ZFMK), 27 ♂♂ “NE-Laos: Hua Phan prov., Ban Saleui, Phou Pan (Mt.) - 20°12'N, 104°01'E; 11.iv.-15.v.2012; 1300–1900m; leg. C. Holzschuh Ankauf ZFMK Bonn 2012” (ZFMK), 13 ♂♂ “Laos-NE, Houa Phan prov., 20°13'09–19''N, 103°59'54''-104°00'03''E, 1480–1510m Phou Pane Mt., 22.IV.–14.V.2008 Vit Kubáň leg. (NMPC), 1 ♂ “Laos-NE, Houa Phan prov., 20°13'09–19''N, 103°59'54''-104°00'03''E, 1480–1510m Phou Pane Mt., 22.IV.–14.V.2008 Vit Kubáň leg.” (NMPC).

##### Description.

**Figure 1.**
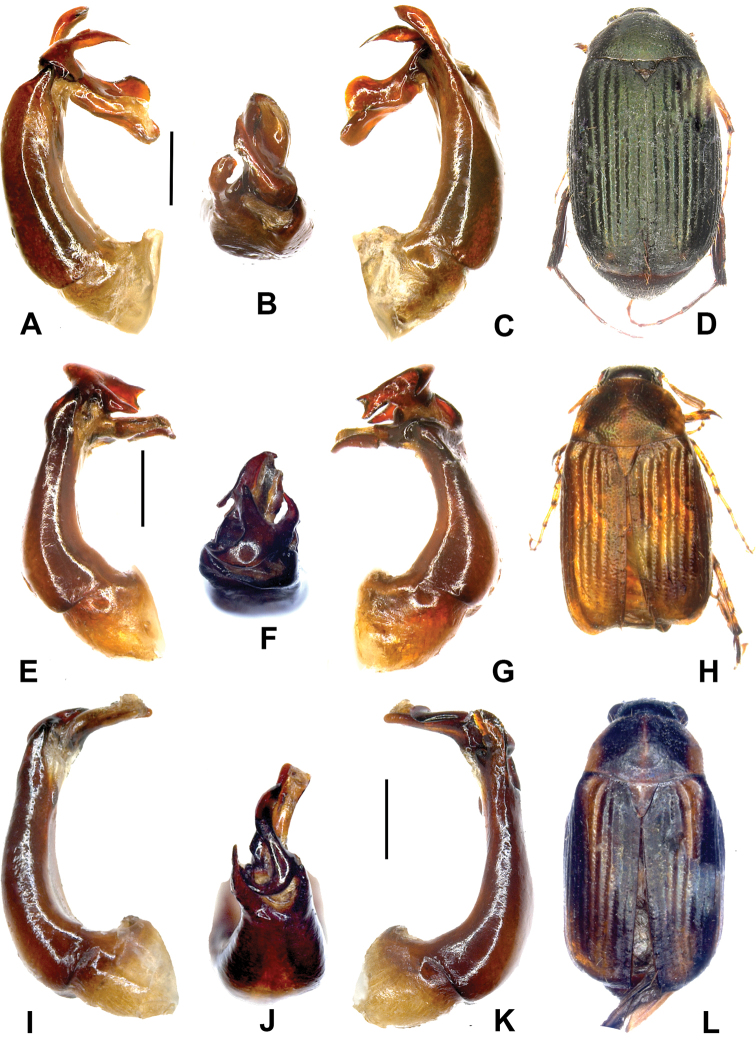
**A–D** *Gastroserica carolusi* sp. n. (holotype) **E–H** *Gastroserica fengduana* (holotype) **I–L** *Gastroserica haoyui* sp. n. (holotype). **A, E, I** Aedeagus, left side lateral view **C, G, K** Aedeagus, right side lateral view; **B, F, J** parameres, dorsal view **D, H, L** Habitus. Scale: 0.5 mm.

Body length: 7.4 mm, length of elytra: 5.9 mm, width: 4.1 mm. Body oval, legs dark brown, elytra and dorsal surface black with greenish shine, with a brown spot on each side of pronotum, dull, with minute, moderately dense setae and sparse, long, erect setae interspersed (Fig. 1D).

Labroclypeus subrectangular and short, widest at middle, lateral margins weakly curved and convergent toward base, anterior angles broadly rounded, lateral border and ocular canthus producing a distinct obtuse angle, anterior margin weakly reflexed, straight, surface moderately convex medially and moderately shiny, coarsely and densely punctate, with several long, erect setae; frontoclypeal suture distinctly impressed and moderately curved, smooth area anterior to eye 1.5 times as wide as long; ocular canthus moderately short and robust, finely and densely punctate, with a short terminal seta. Frons with coarse, dense punctures, irregularly interspersed with fine ones, with sparse, erect setae, impunctate on midline. Eyes moderately large, ratio of diameter / interocular width: 0.71. Antenna brown, with ten antennomeres; club with four antennomeres, slightly longer than remaining antennomeres combined, first joint of club slightly shorter than club. Mentum elevated and flattened anteriorly.

Pronotum moderately wide, widest at base, lateral margins strongly curved and convergent anteriorly, weakly sinuate before posterior angles, anterior angles not produced and strongly rounded, nearly obsolete, posterior angles nearly right-angled, anterior margin almost straight, with distinct and fine marginal line, basal margin moderately curved, without marginal line, base with two shallow impressions on each side at a quarter of width from lateral margin; surface with moderately dense and coarse punctures each bearing a minute seta, not impressed on midline, without transverse impression behind middle; anterior and lateral borders sparsely setose; hypomeron carinate, basal margin of hypomeron strongly produced ventrally. Scutellum subtriangular, apex weakly rounded, with fine and dense punctures, medially smooth, with minute setae.

Elytra oblong, widest at middle, striae distinctly impressed and finely densely punctate; intervals moderately convex, with fine and sparse punctures concentrated along striae, punctures minutely setose, odd intervals with single, coarse punctures each bearing a strong erect seta; epipleural edge moderately strong, ending at strongly convex external apical angle of elytra, epipleura densely setose, apical border chitinous, without short microtrichomes.

Ventral surface dull, with large, dense punctures and dense, short, adpressed setae. Metacoxa partly glabrous, with fine adpressed setae laterally. Abdominal sternites finely and densely punctate, with fine, short setae, each sternite with indistinct transverse row of coarse punctures each bearing a short robust seta. Mesosternum between mesocoxae as wide as mesofemur, with numerous strong setae on an indistinct semicircular carina. Ratio of length of metepisternum / metacoxa: 1 / 1.75. Pygidium long, apically produced and strongly convex, with fine, dense punctures and fine setae, interspersed with a few robust punctures each bearing a robust seta, without smooth midline.

Legs moderately slender and moderately shiny; femora finely densely punctate and densely setose, with two longitudinal rows of longer and more robust setae; anterior edge of metafemur acute lacking an adjacent serrated line, posterior margin weakly convex, with a few fine setae medially, weakly widened in apical half ventrally but not serrate, serrate dorsally, with short setae. Metatibia moderately broad, convexly widened at middle, ratio width / length: 1 / 3, dorsally sharply carinate, with two groups of spines, basal group at one third, apical one at two thirds of metatibial length, with a few single spines in punctures basally; lateral face longitudinally convex, with dense and coarse punctures, some of them longitudinally impressed, ventral edge serrate; medial face impunctate and smooth, apex interiorly near tarsal articulation sharply truncate. Tarsomeres glabrous and finely punctate dorsally, with sparse, short setae ventrally; metatarsomeres with strong longitudinal impressions dorsally, with a strongly serrated ridge ventrally, with a strong longitudinal carina laterally, first metatarsomere shorter than following two tarsomeres combined and twice as long as dorsal tibial spur. Protibia short, bidentate, protarsal claws symmetrical.

##### Aedeagus.

Fig. 1A–C.

##### Diagnosis.

*Gastroserica carolusi* sp. n. is very similar to *Gastroserica mausonensis* Ahrens, 2000, from northern Vietnam; both species may be principally distinguished by shape of parameres: in the new species the lateral process of phallobase is shorter, the ventral process of left paramere is strongly reduced, and the lateral short tooth of the right paramere is displaced behind apical half.

##### Etymology.

The new species is named after one of its collectors, Carous Holzschuh, who donated many Sericini specimens from his Phou Pan collecting to the ZFMK.

##### Variation.

Body length: 7.1–7.8 mm, length of elytra: 5.2–5.9 mm, width: 4.1–4.2 mm. Colour quite variable, from entirely black to yellowish brown with dorsal surface reddish brown with darker frons and pronotal disc, the latter form sometimes with sutural and lateral elytral intervals black, sometimes also 4th interval partly black and the pronotum with a narrow yellow midline. Blackish ventral surface is seen with either reddish or black dorsal surface.

##### Remarks.

The species occurs syntopically with *Gastroserica marginalis* (Brenske, 1894), from which female specimens cannot be distinguished. Therefore, we refrained from assigning female specimens to either species.

#### Gastroserica fengduana

Taxon classificationAnimaliaColeopteraScarabaeidae

Liu & Ahrens sp. n.

http://zoobank.org/44CB8C3C-DD0A-4D96-BAD7-1C135E70B8E0

Figs 1E–H , 4

##### Type material examined.

Holotype ♂ “Fengdu, Shiping, Sichuan, 2.VI.1994, 610m, leg. Zhang Youwei, light trap” (IZAS).

##### Description.

Body length: 7.3 mm, length of elytra: 5.5 mm, width: 4.0 mm. Body oval, including legs yellowish brown, head and disc of pronotum darker, dorsal surface moderately shiny, with sparse, long, erect setae (Fig. 1H).

Labroclypeus subrectangular and short, widest at middle, lateral margins weakly curved, convergent toward base, anterior angles broadly rounded, lateral border and ocular canthus producing a distinct obtuse angle, anterior margin weakly reflexed and straight, surface moderately convex medially and moderately shiny, coarsely and densely punctate, with several long, erect setae; frontoclypeal suture distinctly impressed and moderately curved, smooth area anterior to eye almost twice as wide as long; ocular canthus moderately short and strong, finely and densely punctate, with two terminal setae. Frons with coarse, dense punctures, with fine punctures irregularly interspersed, with dense erect setae. Eyes moderately large (in holotype slightly deformed), ratio of diameter / interocular width: 0.67. Antenna with ten antennomeres; club with four antennomeres, as long as remaining antennomeres combined, first joint of club slightly shorter than club. Mentum elevated and flattened anteriorly.

Pronotum rectangular, widest just before base, lateral margins nearly evenly convergent anteriorly, anterior angles strongly rounded, not produced, nearly obsolete; posterior angles blunt; anterior margin straight, with a distinct, fine marginal line, basal margin moderately curved, without marginal line, base with a shallow impressions on each side beside middle; surface with dense, fine punctures and with minute setae, disc beside midline widely and indistinctly dark, impunctate along midline but not impressed, with a very shallow and indistinct transverse impression behind middle; anterior and lateral borders setose; hypomeron carinate, basal margin of hypomeron strongly produced ventrally. Scutellum subtriangular, apex weakly rounded, with fine and dense punctures, smooth medially, with minute setae.

Elytra oblong, widest at middle, striae distinctly impressed and finely densely punctate; intervals convex, with fine and sparse punctures concentrated along striae, minutely setose in punctures, odd intervals with single coarse punctures each bearing a strong, erect seta; epipleural edge moderately strong, ending at strongly rounded external apical angle of elytra, epipleura densely setose, apical border chitinous, without short microtrichomes.

Ventral surface with large, dense punctures and dense, short adpressed setae. Metacoxa partly glabrous, with fine adpressed setae laterally. Abdominal sternites finely and densely punctate, with fine, short setae, each sternite with indistinct transverse row of coarse punctures each bearing a short robust seta. Mesosternum between mesocoxae as wide as mesofemur, with numerous strong setae. Ratio of length of metepisternum / metacoxa: 1 / 2.0. Pygidium long, apically produced and strongly convex, with fine, dense punctures and fine setae interspersed with few robust punctures each bearing a robust seta, without smooth midline.

Legs moderately slender and shiny; femora finely densely punctate and setose, with two longitudinal rows of setae; anterior edge of metafemur acute, lacking an adjacent serrated line, posterior margin weakly convex, with a few fine setae medially, weakly widened in apical half ventrally but not serrate, serrate dorsally, with short setae. Metatibia moderately broad, convexly widened at middle, ratio width / length: 1 / 3, sharply carinate dorsally, with two groups of spines, basal group at one third, apical one at two thirds of metatibial length, with a few single spines in punctures basally; lateral face longitudinally convex, with moderately dense and fine, minutely setose punctures, some of them longitudinally impressed, ventral edge serrate; medial face impunctate and smooth, apex sharply truncate interiorly near tarsal articulation. Tarsomeres glabrous and finely punctate dorsally, with sparse, short setae ventrally; metatarsomeres with strong longitudinal impressions dorsally, with a strongly serrated ridge ventrally and a strong longitudinal carina laterally, first metatarsomere shorter than following two tarsomeres combined and twice as long as dorsal tibial spur. Protibia short, bidentate, protarsal claws symmetrical.

##### Aedeagus.

Fig. 1E–G.

##### Diagnosis.

The new species is most similar to *Gastroserica sichuana* Ahrens, 2000, in shape of male genitalia and external morphology. It differs distinctly in the median process of the left paramere being shorter, as well as the right paramere being bent ventrally at the apex.

##### Etymology.

Named after the type locality, Fengdu.

#### Gastroserica haoyui

Taxon classificationAnimaliaColeopteraScarabaeidae

Liu & Ahrens sp. n.

http://zoobank.org/23A65ED4-9FFB-4DC2-8D0C-079E686E91D0

Figs 1I–L , 4

##### Type material examined.

Holotype ♂ “Mt. Fengyangshan, Longquan, Zhejiang, 25. VII.-1. VIII. 2007, leg. Liu Haoyu, Gao Zhenhua” (HBUM). Paratype: 1 ♂ “Mts. Fengyangshan, Zhejiang, 31.VII.2008, 1089-1168m, leg. Yang Juan” (IZAS).

##### Description.

Body length: 7.2 mm, length of elytra: 5.6 mm, width: 3.9 mm. Body oval, dark brown, legs, antenna, labroclypeus, midline and lateral margins of pronotum and a moderately wide longitudinal stripe on elytra yellowish brown, dorsal surface moderately shiny, with moderately dense, long, erect setae (Fig. 1L).

Labroclypeus subrectangular and short, widest at middle, lateral margins weakly curved, weakly convergent toward base, anterior angles broadly rounded, lateral border and ocular canthus producing a distinct obtuse angle, anterior margin weakly reflexed, weakly sinuate medially, surface moderately convex medially and moderately shiny, finely and sparsely punctate, with a few coarse punctures, each bearing a long erect seta, interspersed; frontoclypeal suture distinctly impressed and moderately curved, smooth area anterior to eye 1.6 times as wide as long; ocular canthus moderately short and strong, finely and densely punctate, with a fine terminal seta. Frons with coarse, partly very dense punctures, with a few short erect setae beside eyes and behind frontoclypeal suture. Eyes moderately large, ratio of diameter / interocular width: 0.63. Antenna with ten antennomeres; club with four antennomeres, slightly longer than remaining antennomeres combined, first joint of club slightly shorter than club. Mentum elevated and flattened anteriorly.

Pronotum rectangular, widest at base, lateral margins strongly convergent in anterior half, weakly sinuate before posterior angles, anterior angles not produced and strongly rounded, nearly obsolete, posterior angles nearly blunt, anterior margin straight, with a distinct and fine marginal line, basal margin moderately curved, without marginal line; base with a shallow impressions on each side beside middle; surface with dense and fine punctures, with minute setae in punctures, weakly impressed on midline, with a distinct transverse impression behind middle; anterior and lateral borders setose; hypomeron carinate, basal margin of hypomeron strongly produced ventrally. Scutellum subtriangular, apex weakly rounded, with fine and dense punctures, smooth medially, with a few minute setae in punctures.

Elytra oblong, widest at middle, striae distinctly impressed and finely densely punctate; intervals convex, with fine and sparse punctures concentrated along striae, punctures minutely setose, odd intervals with single coarse punctures each bearing a strong erect seta, a light yellowish stripe covers intervals V, III (posterior two thirds), IV, and VI (anterior quarter); epipleural edge moderately strong, ending at strongly rounded external apical angle of elytra, epipleura densely setose, apical border chitinous, without short microtrichomes.

Ventral surface dull, with large, dense punctures and dense, short, adpressed setae. Metacoxa partly glabrous, with fine adpressed setae laterally. Abdominal sternites finely and densely punctate, with fine, short setae, each sternite with indistinct transverse row of coarse punctures each bearing a short strong seta. Mesosternum between mesocoxae as wide as mesofemur, with numerous strong setae. Ratio of length of metepisternum / metacoxa: 1 / 1.71. Pygidium long, apically produced and strongly convex, with fine, dense punctures and fine setae interspersed with a few robust punctures each bearing a robust seta, without smooth midline.

Legs moderately slender and shiny; femora finely, densely punctate and setose, with two longitudinal rows of longer setae; anterior edge of metafemur acute lacking an adjacent serrated line, posterior margin weakly convex, with a few fine setae medially, weakly widened in apical half ventrally but not serrate, serrate dorsally, with short setae. Metatibia moderately broad, convexly widened at middle, ratio width / length: 1 / 3.16, dorsally sharply carinate, with two groups of spines, basal group at one third, apical one at two thirds of metatibial length, with a few single spines in punctures basally; lateral face longitudinally convex, with dense and moderately coarse, longitudinally impressed punctures, ventral edge serrate; medial face impunctate and smooth, apex sharply truncate interiorly near tarsal articulation. Tarsomeres glabrous and finely punctate dorsally, with sparse, short setae ventrally; metatarsomeres with strong longitudinal impressions dorsally, with a strongly serrated ridge ventrally, with a strong longitudinal carina laterally, first metatarsomere slightly shorter than following two tarsomeres combined and twice as long as dorsal tibial spur. Protibia short, bidentate, protarsal claws symmetrical.

##### Aedeagus.

Fig. 1I–K.

##### Diagnosis.

*Gastroserica haoyui* sp. n. differs from all other known *Gastroserica* species by the left paramere being s-shaped.

##### Etymology.

This new species is named after its collector, Liu Haoyu.

##### Variation.

Body length: 7.2 mm, length of elytra: 5.5–5.6 mm, width: 3.9–4.3 mm. Body oval, pale brown.

#### Gastroserica jinxiuensis

Taxon classificationAnimaliaColeopteraScarabaeidae

Liu & Ahrens sp. n.

http://zoobank.org/4B424F15-7F99-45A2-9556-C264A6AE88F9

Figs 2A–D , 4

##### Type material examined.

Holotype ♂ “Yinshanzhan, Jinxiu, Guangxi, 1100m, 10.V.1999, leg. Li Wenzhu” (IZAS).

##### Description.

**Figure 2.**
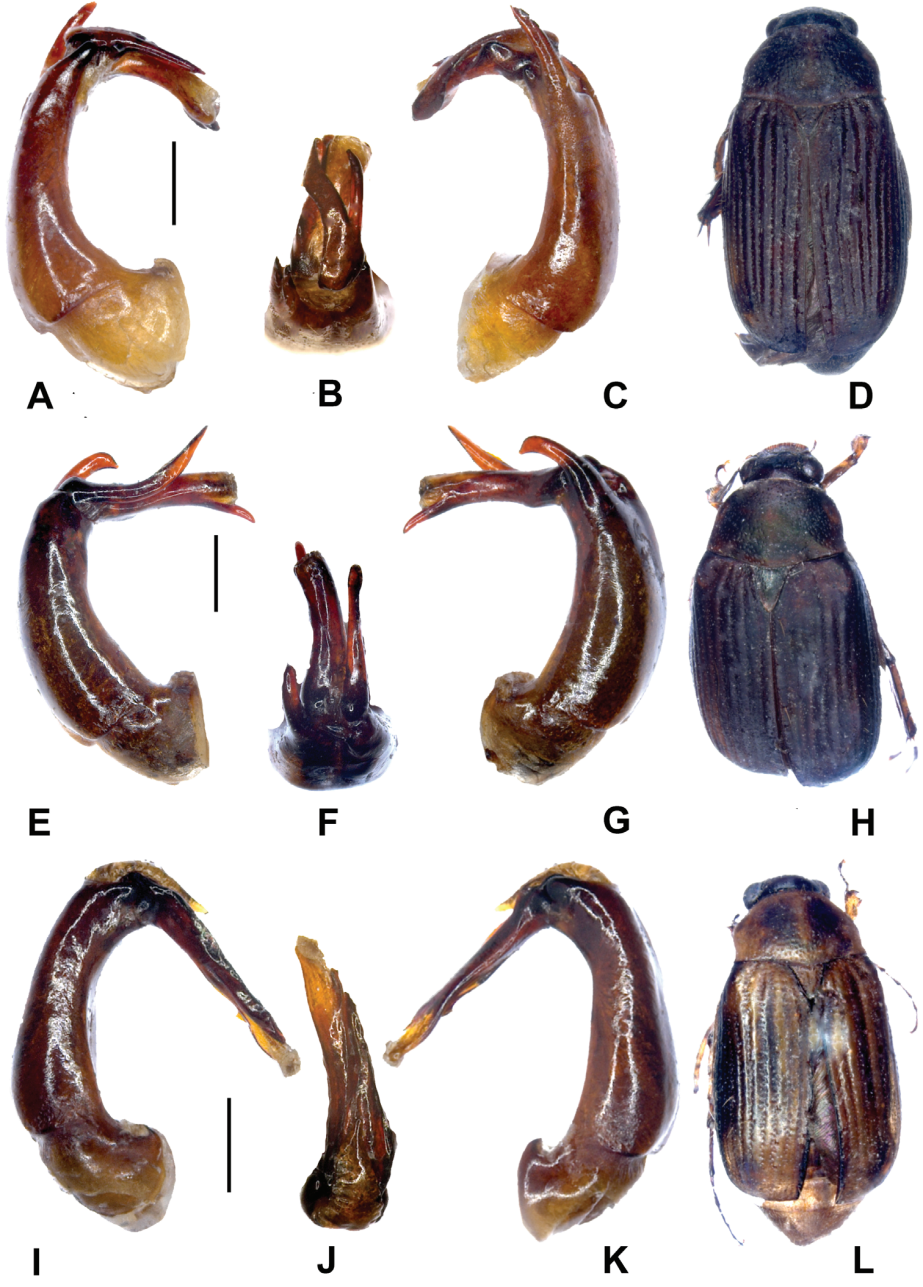
**A–D** *Gastroserica jinxiuensis* sp. n. (holotype) **E–H** *Gastroserica liboensis* (holotype) **I–L** *Gastroserica wenzhui* sp. n. (holotype). **A, E, I** Aedeagus, left side lateral view **C, G, K** Aedeagus, right side lateral view **B, F, J** parameres, dorsal view **D, H, L** Habitus. Scale: 0.5 mm.

Body length: 8.8 mm, length of elytra: 6.7 mm, width: 4.4 mm. Body oval, pronotum, dark brown, surface dull, dorsal surface with moderately dense, long, erect setae (Fig. 2D).

Labroclypeus subrectangular and short, widest at base, lateral margins convex and moderately convergent from base to anterior angles, anterior angles broadly rounded, lateral border and ocular canthus producing a distinct obtuse angle, anterior margin weakly concavely sinuate and weakly reflexed, surface moderately convex medially, moderately shiny, coarsely and sparsely punctate, with several long, erect setae; frontoclypeal suture distinctly impressed and moderately curved, smooth area anterior to eye slightly wider than long; ocular canthus moderately short and strong, finely and densely punctate, terminal seta lacking. Frons coarsely and moderately densely punctate, with fine punctures irregularly interspersed, sparsely setose, most setae abrased in holotype. Eyes moderately large, ratio of diameter / interocular width: 0.52. Antenna missing in holotype. Mentum elevated and flattened anteriorly.

Pronotum rectangular, widest at middle, lateral margins strongly convergent anteriorly, weakly sinuate before posterior angles, anterior angles not produced and strongly rounded, nearly obsolete, posterior angles moderately blunt, anterior margin straight, with a fine marginal line, basal margin moderately curved, without marginal line, base with a weak impression on each side; surface with moderately dense and moderately coarse punctures, punctures with minute setae, moderately impressed on midline, with a weak, transverse impression behind middle; anterior and lateral borders setose; hypomeron carinate, basal margin of hypomeron strongly produced ventrally. Scutellum subtriangular, apex weakly rounded, with fine and dense punctures, smooth medially, with a few minute setae in punctures.

Elytra oblong, widest at middle, striae distinctly impressed and finely densely punctate; intervals weakly convex, with fine and sparse punctures concentrated along striae, punctures minutely setose, odd intervals with single coarse punctures, each bearing a strong erect seta; epipleural edge moderately strong, ending at strongly rounded external apical angle of elytra, epipleura densely setose, apical border chitinous, without short microtrichomes.

Ventral surface with large and dense punctures and dense, short, adpressed setae. Metacoxa partly glabrous, with fine adpressed setae laterally. Abdominal sternites finely and densely punctate, with fine, short setae, each sternite with indistinct transverse row of coarse punctures, each bearing a short strong seta. Mesosternum between mesocoxae as wide as mesofemur, with numerous strong setae. Ratio of length of metepisternum / metacoxa: 1 / 1.79. Pygidium long, apically produced and strongly convex, with fine, dense punctures and fine setae interspersed with a few robust punctures each bearing a robust seta, without smooth midline.

Legs moderately slender and shiny; femora finely densely punctate and setose, with two longitudinal rows of setae; anterior edge of metafemur acute, lacking an adjacent serrated line, posterior margin weakly convex, with a few fine setae medially, weakly widened ventrally in apical half but not serrate, serrate dorsally, with short setae. Metatibia moderately broad, convexly widened at middle, ratio width / length: 1 / 3.33, dorsally sharply carinate, with two groups of spines, basal group at one third, apical one at two thirds of metatibial length, basally with a few single spines in punctures; lateral face longitudinally convex, with moderately dense and fine, longitudinally impressed punctures, ventral edge serrate; medial face impunctate and smooth, apex interiorly near tarsal articulation sharply truncate. Meso and metatarsomeres missing in holotype.

##### Aedeagus.

Fig. 2A–C.

##### Diagnosis.

The species somewhat resembles *Gastroserica kabakovi* Ahrens, 2002, in genital morphology. It differs mainly in the details of paramere shape: in the new taxon, the left paramere is distinctly longer than it is in *Gastroserica kabakovi*, while its two processes are more widely divergent, with the external one reflexed ventrally along its own axis.

##### Etymology.

Named after the type locality, Jinxiu.

#### Gastroserica liboensis

Taxon classificationAnimaliaColeopteraScarabaeidae

Liu & Ahrens sp. n.

http://zoobank.org/82F9A796-D0D6-4828-AA31-B71A7D198C96

Figs 2E–H , 4

##### Type material examined.

Holotype: ♂ “Libo, Guizhou, V.1998, No.1-028, 3-017” (IZAS). Paratype: 1 ♀ “Libo, Guizhou, V.1998, No.1-028, 3-017” (IZAS).

##### Description.

Body length: 8.9 mm, length of elytra: 6.5 mm, width: 4.5 mm. Body oval, dark brown, ventral surface, labroclypeus and sides of pronotum including footstalk of antenna reddish, dorsal surface dull, with moderately dense, long, erect setae (Fig. 2H).

Labroclypeus subrectangular and short, widest at middle, lateral margins weakly curved and convergent towards base, anterior angles broadly rounded, lateral border and ocular canthus producing a distinct obtuse angle, anterior margin weakly reflexed, nearly straight, surface moderately convex medially and moderately shiny, finely and densely punctate, with numerous long, erect setae; frontoclypeal suture distinctly impressed and moderately curved, smooth area anterior to eye almost twice as wide as long; ocular canthus moderately short and strong, finely and densely punctate, with one or two terminal setae. Frons with coarse, dense punctures, with fine punctures irregularly interspersed, with dense erect setae. Eyes moderately large, ratio of diameter / interocular width: 0.68. Antenna with ten antennomeres; club with four antennomeres, as long as remaining antennomeres combined, antennomeres of club subequal in length. Mentum elevated and flattened anteriorly.

Pronotum subtrapezoidal, widest at base, lateral margins strongly convergent in anterior half, almost subparallel in basal half and slightly sinuate before posterior angles, anterior angles not produced and strongly rounded, nearly obsolete, posterior angles moderately sharp, anterior margin almost straight, with a distinct, fine marginal line, basal margin without marginal line; base with a shallow impressions on each side beside middle; surface with moderately dense and moderately coarse punctures, punctures with minute setae, without transverse or longitudinal impressions; anterior and lateral borders setose; hypomeron carinate, basal margin of hypomeron strongly produced ventrally. Scutellum subtriangular, apex weakly rounded, with fine and dense punctures, smooth medially, with a few minute setae in punctures.

Elytra oblong, widest at middle, striae distinctly impressed and finely densely punctate; intervals moderately convex, with fine and sparse punctures concentrated along striae, punctures minutely setose, odd intervals with single coarse punctures each bearing a strong erect seta; epipleural edge moderately strong, ending at strongly rounded external apical angle of elytra, epipleura densely setose, apical border chitinous, without short microtrichomes.

Ventral surface with large and dense punctures and dense, short, adpressed setae. Metacoxa partly glabrous, with fine adpressed setae laterally. Abdominal sternites finely and densely punctate, with fine, short setae, each sternite with indistinct transverse row of coarse punctures each bearing a short robust seta. Mesosternum between mesocoxae as wide as mesofemur, with numerous strong setae. Ratio of length of metepisternum / metacoxa: 1 / 1.78. Pygidium long, apically produced and strongly convex, with fine, dense punctures and fine setae interspersed with a few robust punctures each bearing a robust seta, without smooth midline.

Legs moderately slender and shiny; femora finely densely punctate and setose, with two longitudinal rows of setae; anterior edge of metafemur acute, lacking an adjacent serrated line, posterior margin weakly convex, with a few fine setae medially, weakly widened in apical half ventrally but not serrate, serrate dorsally, with short setae. Metatibia moderately broad, convexly widened at middle, ratio width / length: 1 / 3.0, dorsally sharply carinate, with two groups of spines, basal group at one third, apical one at two thirds of metatibial length, with a few single spines in punctures basally; lateral face longitudinally convex, with dense and fine, longitudinally impressed punctures, ventral edge serrate; medial face impunctate and smooth, apex interiorly near tarsal articulation sharply truncate. Metatarsomeres missing in holotype.

##### Aedeagus.

Fig. 2E–G.

##### Diagnosis.

The new species is, to some extent, similar to *Gastroserica angustula* Brenske, 1897, in genital morphology, but differs in being slightly larger in body size and in having the lateral apical apophysis of phallobase shorter, the right paramere lacking a small lateral tooth, and the left paramere being curved dorsally.

##### Etymology.

Named after the type locality, Libo.

##### Variation.

Female: Body length: 9.1 mm, length of elytra: 6.8 mm, width: 4.5 mm. Body oval, reddish brown, antenna pale brown. Eyes small, ratio of diameter / interocular width: 0.58. Ratio of length of metepisternum / metacoxa: 1 / 1.91. Metatibia moderately broad, convexly widened at middle, ratio width / length: 1 / 3.2. Tarsomeres glabrous and finely punctate dorsally, with sparse, short setae ventrally; metatarsomeres with strong longitudinal impressions dorsally, with a strongly serrated ridge ventrally, with a strong longitudinal carina laterally, first metatarsomere as long as following two tarsomeres combined and twice as long as ventral tibial spur.

#### Gastroserica wenzhui

Taxon classificationAnimaliaColeopteraScarabaeidae

Liu & Ahrens sp. n.

http://zoobank.org/DEA6168E-120A-4FED-8060-98BC815B8FC5

Figs 2I–L , 4

##### Type material examined.

Holotype: ♂ “Defu, Napo, Guangxi, 1350m, 18.VI.2000, leg. Li Wenzhu” (IZAS). Paratypes: 1 ♂ “Fulong, Fangcheng City, Guangxi, 500m, 24.V.1999, leg. Liu Dajun” (IZAS), 1 ♀ “Fulong, Fangcheng City, Guangxi, 24.V.1999, 500m, leg. Liu Dajun” (IZAS).

##### Description.

Body length: 7.8 mm, length of elytra: 6.0 mm, width: 3.9 mm. Body oval, yellowish brown, frons, two large maculae and 4th and lateral intervals of elytra as well as abdomen darker, dorsal surface moderately shiny, frons and disc of pronotum with some greenish sheen, with moderately dense, long, erect setae (Fig. 2L).

Labroclypeus subellyptical, widest at middle, lateral margins convex, strongly convergent toward base and to broadly rounded anterior angles, lateral border and ocular canthus producing a distinct obtuse angle, anterior margin weakly reflexed, straight, surface moderately convex medially and moderately shiny, coarsely and densely punctate, with several long, erect setae; frontoclypeal suture distinctly impressed and moderately curved, smooth area anterior to eye 1.3 times as wide as long; ocular canthus moderately short and strong, finely and densely punctate, with two terminal setae. Frons with coarse, dense punctures and with fine, dense punctures regularly interspersed, covered with dense erect setae. Eyes moderately large, ratio of diameter / interocular width: 0.63. Antenna with ten antennomeres; club with four antennomeres, slightly longer than remaining antennomeres combined. Mentum elevated and flattened anteriorly.

Pronotum moderately wide, widest at base, lateral margins strongly convergent in anterior half, subparallel in basal half and weakly sinuate before posterior angles, anterior angles not produced and strongly rounded, nearly obsolete, posterior angles moderately sharp, anterior margin straight, with a distinct, fine marginal line, basal margin without marginal line; surface with dense and moderately fine punctures, punctures with minute setae, without impressions; anterior and lateral borders setose; hypomeron carinate, basal margin of hypomeron strongly produced ventrally. Scutellum subtriangular, apex weakly rounded, with fine and dense punctures, medially smooth, with a few minute setae in punctures.

Elytra oblong, widest at posterior third, striae distinctly impressed and finely densely punctate; intervals moderately convex, with fine and sparse punctures concentrated along striae, punctures minutely setose, odd intervals with single coarse punctures, each bearing a strong erect seta, sutural and 4th interval as well as lateral two intervals blackish; epipleural edge moderately strong, ending at strongly rounded external apical angle of elytra, epipleura densely setose, apical border chitinous, without short microtrichomes.

Ventral surface with large and dense punctures and dense, short, adpressed setae. Metacoxa partly glabrous, with fine adpressed setae laterally. Abdominal sternites finely and densely punctate, with fine, short setae, each sternite with indistinct transverse row of coarse punctures, each bearing a short strong seta. Mesosternum between mesocoxae as wide as mesofemur, with numerous strong setae. Ratio of length of metepisternum / metacoxa: 1 / 2.0. Pygidium long, apically produced and strongly convex, with fine, dense punctures and fine setae interspersed with a few robust punctures each bearing a robust seta, without smooth midline.

Legs moderately slender and shiny; femora finely densely punctate and setose, with two longitudinal rows of setae; anterior edge of metafemur acute lacking an adjacent serrated line, posterior margin weakly convex, with a few fine setae medially, weakly widened ventrally in apical half but not serrate, serrate dorsally, with short setae. Metatibia moderately broad, convexly widened at middle, ratio width / length: 1 / 3.33, dorsally sharply carinate, with two groups of spines, basal group at one third, apical one at two thirds of metatibial length, with a few single spines in punctures basally; lateral face longitudinally convex, with dense and fine, longitudinally impressed punctures, ventral edge serrate; medial face impunctate and smooth, apex interiorly near tarsal articulation sharply truncate. Tarsomeres glabrous and finely punctate dorsally, with sparse, short setae ventrally; metatarsomeres with strong longitudinal impressions dorsally, with a strongly serrated ridge ventrally, with a strong longitudinal carina laterally, first metatarsomere as long as following two tarsomeres combined and twice as long as dorsal tibial spur. Protibia short, bidentate, protarsal claws symmetrical.

##### Aedeagus.

Fig. 2I–K.

##### Diagnosis.

The new species is very similar to *Gastroserica pickai* Ahrens, 2000, from northern Vietnam in external and genital morphology. The new taxon differs from *Gastroserica pickai* in having much more narrow parameres.

##### Etymology.

The new species is named after one of its collectors, Li Wenzhu.

##### Variation.

Body length: 7.1–7.8 mm, length of elytra: 5.3–6.0 mm, width: 3.9–4.1 mm. Female: smooth area anterior to eye as wide as long; club with three antennomeres, as long as remaining antennomeres combined.

#### Gastroserica damingshanica

Taxon classificationAnimaliaColeopteraScarabaeidae

Liu & Ahrens sp. n.

http://zoobank.org/D4B1F8CB-7B47-4BD7-A61F-F1C448A91F24

Figs 3A–D , 4

##### Type material examined.

Holotype. ♂ “Guangxi, Damingshan, 20.V.2011, 1230m, 23°30'N, 108°26'E, leg. Zhang Youwei” (IZAS).

##### Description.

**Figure 3.**
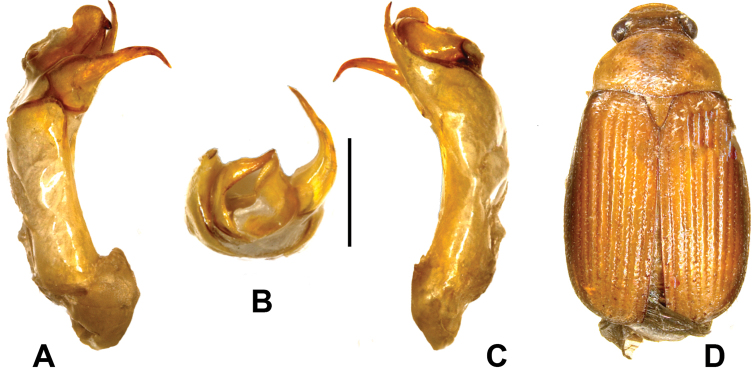
**A–D** *Gastroserica damingshanica* sp. n. (holotype) **A** Aedeagus, left side lateral view **C** Aedeagus, right side lateral view **B** parameres, dorsal view **D** Habitus. Scale: 0.5 mm.

Body length: 6.7 mm, length of elytra: 4.7 mm, width: 3.8 mm. Body oval, pronotum and ventral surface yellowish brown, frons and two spots on disc of pronotum darker, elytra reddish brown, lateral intervals darker, abdomen dark brown to grey. Dorsal surface moderately shiny, with sparse setae (Fig. 3D).

Labroclypeus subelliptical, widest at middle, lateral margins strongly convex and convergent toward base, anterior angles broadly rounded, lateral border and ocular canthus producing a distinct obtuse angle, anterior margin weakly reflexed and straight, surface moderately convex medially and moderately shiny, coarsely and densely punctuate; frontoclypeal suture weakly impressed and moderately curved, smooth area anterior to eye almost 1.3 times as wide as long; ocular canthus moderately long and narrow, finely and densely punctate, without terminal seta. Frons with a few coarse and fine punctures, with a few short setae behind frontoclypeal suture and on posterior portion. Eyes moderately large, ratio of diameter / interocular width: 0.65. Antenna with ten antennomeres; club with four antennomeres, as long as remaining antennomeres combined. Mentum elevated and flattened anteriorly.

Pronotum rectangular, widest at base, lateral margins nearly evenly convergent anteriorly, slightly concavely sinuate before posterior angles, anterior angles strongly rounded, not produced, nearly obsolete, posterior angles blunt, anterior margin straight, with a distinct, fine marginal line, basal margin moderately curved, without marginal line; surface with dense and fine punctures and with minute, adpressed setae; anterior border sparsely setose, setae of lateral margin lacking; hypomeron carinate, basal margin of hypomeron moderately produced ventrally. Scutellum subtriangular, apex weakly rounded, with fine and dense punctures, smooth medially, with minute setae.

Elytra oblong, widest at middle, striae distinctly impressed and finely densely punctate; intervals convex, with fine and sparse punctures concentrated along striae, minutely setose in punctures, odd intervals with larger, single punctures whose setae are abraded, however, lateral intervals with a few setae remaining; epipleural edge moderately strong, ending at strongly rounded external apical angle of elytra, epipleura densely setose, apical border chitinous, without short microtrichomes.

Ventral surface with large, dense punctures and dense, short adpressed setae. Metacoxa setose, with several strong adpressed setae laterally. Abdominal sternites finely and densely punctate, with fine, short setae, each sternite with indistinct transverse row of coarse punctures each bearing a short robust seta. Mesosternum between mesocoxae as wide as mesofemur, with numerous strong setae. Ratio of length of metepisternum / metacoxa: 1 / 1.93. Pygidium long, apically produced and strongly convex, with fine, dense punctures and fine setae interspersed with a few robust punctures each bearing a robust seta, without smooth midline.

Legs moderately strong and shiny; femora finely densely punctate and setose, with two longitudinal rows of setae; anterior edge of metafemur acute lacking an adjacent serrated line, posterior margin weakly convex, with a few fine setae medially, weakly widened in apical half ventrally but not serrate, serrate dorsally, with short setae. Metatibia moderately broad, convexly widened at middle, ratio width / length: 1 / 3, dorsally sharply carinate, with two groups of spines, basal group at one third, apical one at two thirds of metatibial length, with a few single spines in punctures basally; lateral face longitudinally convex, with moderately dense and fine, minutely setose punctures, some of them longitudinally impressed, ventral edge serrate; medial face impunctate and smooth, apex interiorly near tarsal articulation sharply truncate. Tarsomeres missing on holotype.

##### Aedeagus.

Fig. 3A–C.

##### Diagnosis.

The new species is most closely related to *Gastroserica nikodymi* Ahrens, 2000, in external appearance and genital morphology. It differs significantly in the shape of the right lateral process of phallobase, which is sharply pointed apically; the parameres are quite different: the left one is longer and evenly curved ventrally, the right one is lacking a basal lobe.

##### Etymology.

The new species is named after the type locality, Damingshan Mountains.

**Figure 4.**
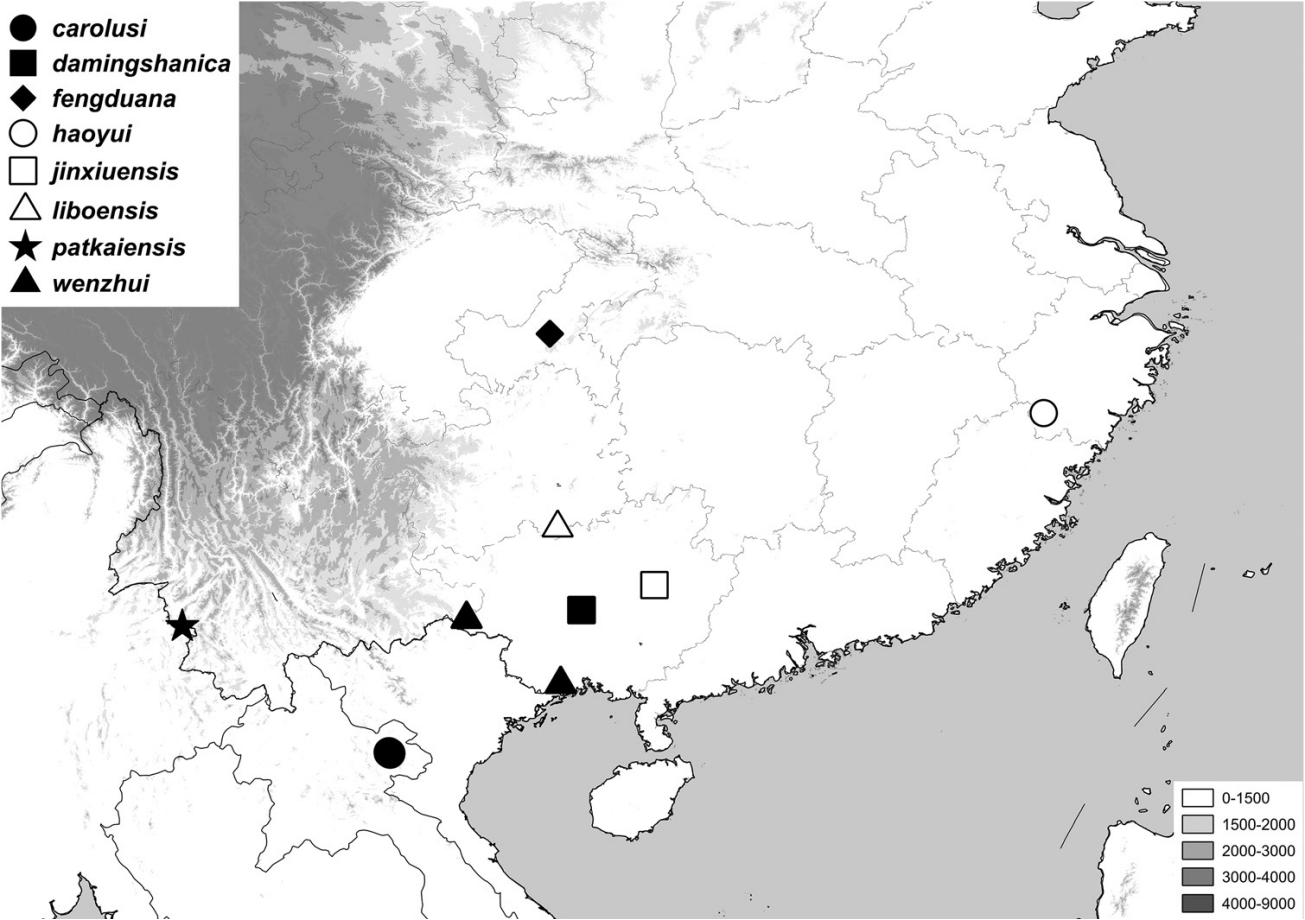
Distribution of newly described *Gastroserica* species.

### New records:

#### Gastroserica asulcata

Taxon classificationAnimaliaColeopteraScarabaeidae

Ahrens, 2000

##### Material examined.

1 ♂ “Mts. Leigongshan, Leishan, Guizhou, 30.VI.1988, 1550m, leg. Wang Shuyong” (IZAS), 1 ♂ “Mts. Tianpingshan, Longsheng, Guangxi, 9.VI.1963, 740m, leg. Wang Shuyong” (IZAS), 1 ♂ “Taiyuan, Pengshui, Sichuan, 10.VII.1989, 850m, leg. Yang Longlong” (IZAS), 1 ♂ “Baiyan, Longsheng, Sichuan, 23.VI.1963, 1150m, leg. Wang Shuyong” (IZAS), 1 ♂ “Mts. Jiulianshan, Jiangxi, 20.VI.1979, 850m, leg. Zhang Youwei” (IZAS). 1 ♂ “Mts. Leigongshan, Leishan, Guizhou, 1.VII.1988, 1050m, leg. Wang Shuyong” (IZAS).

#### Gastroserica fanjingensis

Taxon classificationAnimaliaColeopteraScarabaeidae

Ahrens, 2000

##### Material examined.

3 ♂♂ “Taiyuan, Pengshui, Sichuan, 10,12.VII.1989, 800,850m, leg. Yang Longlong” (IZAS), 2 ♂♂ “Mts. Fanjingshan, Jiangkou, Guizhou, 12.VIII.1988, 530m, leg. Wang Shuyong” (IZAS), 1 ♂ “Mts. Tianpingshan, Longsheng, Guangxi, 4.VI.1963, 740m, leg. Shi Yongshan” (IZAS), 6 ex. “CH-Ghuizhou NE 27.V.–3.VI. 20 km NW of Jiankou 1995 Fanjing Shan- Kuaichang E. Jendek & O. Šauša” (CP).

#### Gastroserica guangdongensis

Taxon classificationAnimaliaColeopteraScarabaeidae

Ahrens, 2000

##### Material examined.

1 ♂ “Mts. Chebaling, Shixing, Guangdong, 25.IV.1991, leg. Li Fasheng” (CAU), 1 ♂ “Huangxizhou, Mts. Wuyishan, Fujian, 27.V.2004, leg. Yuan Caixia, Li Jing” (HBUM), 8 ex. “China: Fujian Province; Mt. Liang-shan-ding, Wuping County, July 2–13, 2009 local collectors Ankauf via Li Jingke 2010” (ZFMK).

#### Gastroserica guizhouana

Taxon classificationAnimaliaColeopteraScarabaeidae

Ahrens, 2000

##### Material examined.

1 ♂ “Mts. Tianping, Longsheng, Guangxi, 4.VI.1963, 740m, leg. Wang Shuyong” (IZAS), 1 ♂ “Qiaoting, Wan County, Sichuan, 27.VI.1974, 1800m, leg. Han Yinheng” (IZAS), 1 ♂ “Mts. Shengtangshan, Jinxiu, Guangxi, 18.V.1999, leg. Yang Xingke” (IZAS), 2 ♂♂ “Mts. Tianpingshan, Longsheng, Guangxi, 5.VI.1963, leg. Shi Yongshan” (IZAS).

#### Gastroserica haucki

Taxon classificationAnimaliaColeopteraScarabaeidae

Ahrens, 2000

##### Material examined.

1 ♂ “Menghun, Xishuangbanna, Yunnan, 1200–1400 m, 22.5.1958, Zhang Yiran leg.” (IZAS), 1 ♀ “Menghun, Xishuangbanna, Yunnan, 1200–1400 m, 22.5.1958, Zhang Yiran leg.” (IZAS), 1 ♂ “NE-Laos: Hua Phan prov., Ban Saleui, Phou Pan (Mt.) ~20°12'N, 104°01'E; 1300–1900m; 01.–31.V.2011; leg. C. Holzschuh Ankauf ZFMK Bonn 2011” (ZFMK), 2 ♂♂, 3 ♀♀ “NE-Laos: Hua Phan prov., Ban Saleui, Phou Pan (Mt.) - 20°12'N, 104°01'E; 14.iv.–15.v.2012; 1300–1900m; leg. C. Holzschuh Ankauf ZFMK Bonn 2012/13” (ZFMK), 1 ♀ “Phu Rua N.P. (900m alt.) Loei P. NE Thai. 26–30.IV.2006 Takakuwa, M. leg.” (ZFMK), 1 ♂, 6 ♀♀ “Laos-NE, Houa Phan prov., 20°13'09–19''N, 103°59'54''-104°00'03''E, 1480–1510m Phou Pane Mt., 22.IV.–14.V.2008 Vit Kuban leg. (NMPC, ZFMK), 1 ♀ “Laos-NE Hua Phan prov., 20°12'N, 104°01'E, Phu Phan Mt., 1500–1900m, 17.5.–3.6.2007, leg. Vit Kuban” (ZFMK).

#### Gastroserica herzi

Taxon classificationAnimaliaColeopteraScarabaeidae

(Heyden, 1887)

##### Material examined.

1 ♂ “Aotou, Huangkeng, Jianyang, Fujian, 5.V.1960, 800–950m, leg. Pu Fuji” (IZAS).

#### Gastroserica huaphanensis

Taxon classificationAnimaliaColeopteraScarabaeidae

Ahrens & Pacholátko, 2003

##### Material examined.

2 ♂♂ “Laos-NE, Houa Phan prov., 20°13'09–19''N, 103°59'54''-104°00'03''E, 1480–1510m Phou Pane Mt., 22.IV.–14.V.2008 Vit Kuban leg. (NMPC, ZFMK), 175 ex. “NE-Laos: Hua Phan prov., Ban Saleui, Phou Pan (Mt.) - 20°12'N, 104°01'E; 14.iv.–15.v.2012; 1300–1900m; leg. C. Holzschuh Ankauf ZFMK Bonn 2012/13” (ZFMK), 7 ex. “NE-Laos: Hua Phan prov., Ban Saleui, Phou Pan (Mt.) ~20°12'N, 104°01'E; 1300–1900m; 01.-31.V.2011; leg. C. Holzschuh Ankauf ZFMK Bonn 2011” (ZFMK), 39 ex. “NE-Laos: Hua Phan prov., Ban Saleui, Phou Pan (Mt.) - 20°12'N, 104°01'E; 11.iv.-15.v.2012; 1300–1900m; leg. C. Holzschuh Ankauf ZFMK Bonn 2012” (ZFMK), 1 ex. “Laos-NE Hua Phan prov., 20°12'N, 104°01'E, Phu Phan Mt., 1500–1900m, 17.5.–3.6.2007, leg. C. Holzschuh” (ZFMK), 2 ex. “Laos-NE, Houa Phan prov., 20°13'09–19''N, 103°59'54''-104°00'03''E, 1480–1510m Phou Pane Mt., 22.IV.–14.V.2008 Vit Kubáň leg. (NMPC, ZFMK).

#### Gastroserica hubeiana

Taxon classificationAnimaliaColeopteraScarabaeidae

Ahrens, 2000

##### Material examined.

2 ♂♂ “Longmenhe River, Xingshan, Hubei, 1300m, 10.5.1994, Zhang Youwei leg.” (IZAS), 1 ♂ “Xinmaopeng, Mt. Tianmu Shan, Zhejiang, 1300m, 28.6.1957, collector unknown” (IZAS), 1 ♂ “Qingyin’ge, Mt. Emei Shan, Sichuan, 800–1000m, 28.5. 1957, Wang Zongyuan leg.” (IZAS).

#### Gastroserica impressicollis

Taxon classificationAnimaliaColeopteraScarabaeidae

(Fairmaire, 1891)

##### Material examined.

1 ♂ “Qiaoting, Wan County, Sichuan, 27.VI.1974, 1300m, leg. Han Yinheng” (IZAS), 1 ♂, 1 ♀ “Ku-ling, 7,11.VII.1935, leg. O. Piel, Musee Heude” (IZAS), 1 ♂ “Mts. Tienmushan, 11.VI.1936, leg. O. Piel, Musee Heude” (IZAS).

#### Gastroserica kabakovi

Taxon classificationAnimaliaColeopteraScarabaeidae

Ahrens, 2002

##### Material examined.

1 ♂ “[China] Yunnan, Mt. Fofangshan, 2010-7-27, 22.59306N, 99.99068E, 1680m/ LW-1313” (IZAS).

##### Remark.

This species was originally described from northern Vietnam; this is the first record for China.

#### Gastroserica kucerai

Taxon classificationAnimaliaColeopteraScarabaeidae

Ahrens & Pacholátko, 2003

##### Material examined.

1 ♂ “Mts. Qingchengshan, Sichuan, 2.VII.1979, 3100m, leg. Shang Jinwen” (IZAS), 1 ♂ “Tianshidong, Mts. Qingchengshan, Sichuan, 5.VI.1979, 1000m, leg. Gao Ping” (IZAS), 1 ♂ “Qinghe Forestry Farm, Kang County, Gansu, 8.VII.1999, 1400m, leg. Yao Jian” (IZAS), 1 ♂ “Jinzhong Road, Jinxiu, Guangxi, 12.V.1999, 1000m, leg. Gao Mingyuan” (IZAS), 1 ♂ “Baoguosi Temple, Mts. Emeishan, Sichuan, 11.V.1957, 550–750m, leg. Lu Youcai” (IZAS), 1 ♀ “Baoguosi Temple, Mts. Emeishan, Sichuan, 15.VI.1957, 800–1000m, leg. Huang Keren” (IZAS).

#### Gastroserica marginalis

Taxon classificationAnimaliaColeopteraScarabaeidae

(Brenske, 1894)

##### Material examined.

1 ♂ “Mts. Jiulianshan, Jiangxi, 21.VI.1975, Zhang Youwei leg.” (IZAS), 1 ♂ “Mt. Jianfengling, Hainan, 800–1000m, 19.V.1997, leg. Yu Peiyu” (IZAS), 1 ♂ “Shiping, Fengdu, Sichuan, 610m, 2.VI.1994, Zhang Youwei leg.” (IZAS), 1 ♂ “Mts. Tianpingshan, Longsheng, Guangxi, 4.VI.1963, 740m, leg. Shi Yongshan” (IZAS), 1 ♂ “Muyuping, Shennongjia, Hubei, 4.VII.1981, 1200m, leg. Han Yinheng” (IZAS), 6 ex. “China: Fujian Province; Mt. Liang-shan-ding, Wuping County, July 2–12, 2009 local collectors Ankauf via Li Jingke 2010” (ZFMK), 33 ex. “Laos-NE, Houa Phan prov., 20°13'09–19''N, 103°59'54''-104°00'03''E, 1480–1510m Phou Pane Mt., 22.IV.–14.V.2008 Vit Kubáň leg.” (NMPC, ZFMK), 217 ex. “NE-Laos: Hua Phan prov., Ban Saleui, Phou Pan (Mt.) - 20°12'N, 104°01'E; 14.iv.–15.v.2012; 1300–1900m; leg. C. Holzschuh Ankauf ZFMK Bonn 2012/13” (ZFMK), 48 ex. “NE-Laos: Hua Phan prov., Ban Saleui, Phou Pan (Mt.) - 20°12'N, 104°01'E; 11.iv.-15.v.2012; 1300–1900m; leg. C. Holzschuh Ankauf ZFMK Bonn 2012” (ZFMK), 6 ex. “Laos-NE Hua Phan prov., 20°12'N, 104°01'E, Phu Phan Mt., 1500–1900m, 17.5.–3.6.2007, leg. C. Holzschuh” (ZFMK), 9 ex. “Laos-NE Hua Phan prov., 20°12'N, 104°01'E, Phu Phan Mt., 1500–1900m, 17.5.–3.6.2007, leg. Vit Kuban” (ZFMK), 1 ♂ “Myanmar, Mandalay prov., Kyaukpadanng, 10.6.2009 From Li Jingke” (ZFMK).

#### Gastroserica nigrofasciata

Taxon classificationAnimaliaColeopteraScarabaeidae

Liu, Ahrens, Bai & Yang, 2011

##### Material examined.

1 ♂ “Sangang, Chonganxingcun, Fujian, 17.V.1960, 740–900m, leg. Ma Chenglin” (IZAS), 1 ♂ “Luoxiang, Jinxiu, Guangxi, 1.VII.2000, 450m, leg. Chen Jun” (IZAS), 1 ♂, 2 ♀♀ “Mts. Zuohushan, Longsheng, Guangxi, 23.VI.1982, leg. Yang Jikun”(CAU).

#### Gastroserica nikodymi

Taxon classificationAnimaliaColeopteraScarabaeidae

Ahrens, 2000

##### Material examined.

2 ex. “China, Fujian c., 21.-22.VI. Ziyungdongshan, NW slopes 25°46'N, 117°20'E, 900–1100m Jaroslav Turna leg., 2007” (CA).

#### Gastroserica patkaiensis

Taxon classificationAnimaliaColeopteraScarabaeidae

Ahrens, 2000

##### Material examined.

1 ♂ “Cangyuan County, Yunnan, 16.V.1980, 990m, leg. Shang Jinwen” (IZAS).

##### Remark.

First record for China.

#### Gastroserica sichuana

Taxon classificationAnimaliaColeopteraScarabaeidae

Ahrens, 2000

##### Material examined.

1 ♂ “Qingyin Ge, Mts. Emeishan, Sichuan, 11.VI.1957, 800–1000m, leg. Lu Youcai” (IZAS), 1 ♂ “Qingyinge, Mts. Emeishan, Sichuan, 25.VI.1957, 800–1000m, leg. Huang Keren” (IZAS).

#### Gastroserica yingi

Taxon classificationAnimaliaColeopteraScarabaeidae

Ahrens & Pacholátko, 2007

##### Material examined.

1 ♂ ”Huawangshanzhuang, Jinxiu, Guangxi, 900m, 20.V.1999, Zhang Yanzhou leg.” (IZAS), 1 ♂ ”Tonkin, Mont Bavi, 900–1000m, VIII.1940, leg. P.A. de Cooman” (IZAS), 1 ♂”Mengzhe, Xishuangbanna, Yunnan, 1200m, 15.VI.1958, Pu Fuji leg.” (IZAS), 1 ♂ “Tianchi, Jianfeng, Hainan, 11.IV.1980, 900m” (IZAS).

##### Remarks.

The specimens examined here, except the one from Tonkin, diverge somewhat from the holotype of *Gastroserica yingi* in the shape of the right paramere. However, it cannot be determined if this is due to local variation, or if there is some as yet unrecognized geographical pattern.

#### Gastroserica yunnanensis

Taxon classificationAnimaliaColeopteraScarabaeidae

Liu, Ahrens, Bai & Yang, 2011

##### Material examined.

1 ♂ “Caiyang River Nature Preserve, Pu’er, Yunnan, 28–29.8.2007, Shi Lei leg.” (LSSYU).

## Supplementary Material

XML Treatment for Gastroserica carolusi

XML Treatment for Gastroserica fengduana

XML Treatment for Gastroserica haoyui

XML Treatment for Gastroserica jinxiuensis

XML Treatment for Gastroserica liboensis

XML Treatment for Gastroserica wenzhui

XML Treatment for Gastroserica damingshanica

XML Treatment for Gastroserica asulcata

XML Treatment for Gastroserica fanjingensis

XML Treatment for Gastroserica guangdongensis

XML Treatment for Gastroserica guizhouana

XML Treatment for Gastroserica haucki

XML Treatment for Gastroserica herzi

XML Treatment for Gastroserica huaphanensis

XML Treatment for Gastroserica hubeiana

XML Treatment for Gastroserica impressicollis

XML Treatment for Gastroserica kabakovi

XML Treatment for Gastroserica kucerai

XML Treatment for Gastroserica marginalis

XML Treatment for Gastroserica nigrofasciata

XML Treatment for Gastroserica nikodymi

XML Treatment for Gastroserica patkaiensis

XML Treatment for Gastroserica sichuana

XML Treatment for Gastroserica yingi

XML Treatment for Gastroserica yunnanensis
